# Hospital Meetings, &c.

**Published:** 1900-07-07

**Authors:** 


### The Institutional Workshop.

### HOSPITAL MEETINGS, &C.

### ST. BARTHOLOMEW'S HOSPITAL.

The Prince of Wales presided on the 2nd inst. over a special general court of governors of St. Bartholomew's Hospital to consider a scheme for the proposed acquisition by that institution of a portion of the site of Christ's Hospital, coupled with that scheme being an alternative proposal by the medical staff that the whole of the site, five acres in extent, should be acquired, instead of only a portion thereof.

According to the report, which appears in the *Times*, after some formal business had been transacted, His Royal Highness called on Sir Trevor Lawrence, the treasurer, to make his statement.

Sir Trevor Lawrence: May it please your Royal Highness, my Lord Mayor, and Gentlemen,—I think in the first place you must allow me, as representing the governors of the hospital, to say one word of thanks to your Royal Highness for your great kindness in coming amongst us to-day. (Cheers.) I can assure the governors of this hospital that I have had numerous instances showing the very great interest which His Royal Highness has been kind enough to take in the affairs of this institution, and, having regard to the many very urgent claims on His Royal Highness's time, I venture to think that we ought to be very grateful to him for coming here to-day. (Cheers.) I think you will agree with me that we have arrived at a somewhat critical stage in the history of this hospital. I venture to think that the question which we have before us to-day is an important public question, not one affecting the hospital only, but one of far-reaching importance. This is the only hospital within the limits of the City of London. It is not every governor who is aware that it was founded in the year 1123, in the twenty-third year of the reign of His Royal Highness's predecessor, Henry I. Consequently it has been carrying on its work of mercy and charity for the greater part of 800 years. This hospital is in reality the creation, the child, of the citizens of London. There are on its walls innumerable records of the gifts and bequests to it which bear testimony to the affection which the citizens of London have ever shown to this hospital, reaching back to 1547. If you look for one moment at the work which is being done by the hospital it is impossible for even the most careless individual to shut his eyes to its importance. In the last ten years we treated here 64,000 in-patients, 158,000 out-patients, 1,385,000 casualty patients, and 17,000 maternity patients, making a total in the ten years of 1,624,000, or 162,000 per annum. That being so, the importance of the work cannot be doubted. At the same time, it has been found that the area at the disposal of the governors of this hospital for carrying on the work has become more and more insufficient. The work has become more and more hampered, and it has gradually become more and more impossible to meet the requirements of modern science. In the report which has been read there is a very interesting letter from the medical staff, and they call attention to the urgent necessity of enlarging the boundary of the hospital. There are special departments required for the eye, for the skin, for the ear, for dental surgery, for the throat, and for electric treatment. A new casualty department is urgently required, and a new out-patient department. A new nursing home is also most urgently required. Unfortunately, our nurses, a most valuable body of women, are scattered about, some here and some there, and there is no proper arrangement for their living in comfort. A new pathological laboratory is required, a new mortuary, a new isolation block, new operating theatres, and a new college and quarters for the resident medical staff. That enumeration shows how urgently more land is required if the hospital is to carry on its work as it has done in the past. We have here a map which sets out the present position of the land of Christ's Hospital and St. Bartholomew's Hospital. The red portion is St. Bartholomew's Hospital and the blue portion is Christ's Hospital. The land which we absolutely require and cannot do without under any circumstances whatever is the land on the upper side of the red line. It is somewhat remarkable that in the year 1819 Christ's Hospital found itself in a position of very considerable embarrassment, and it applied to us to sell them some land. We sold them the land. Their only frontage in Newgate Street consists of a portion of land which we sold to them for their convenience. The map also shows that it is absolutely impossible for the limits of this hospital to be extended in any direction but one. That conical-shaped piece of land which is hospital land is bounded on the north by Smithfield, on the east by the streets that run down King Edward Street, on the west by Giltspur Street, and on the other side by Christ's Hospital; so that it is absolutely impossible to enlarge our boundaries in any direction except in the direction of Christ's Hospital. I should like for a moment to call the attention of the governors to the fact that the land without which we cannot possibly carry on the work of the hospital is all back land. There is only a small amount of frontage in Giltspur Street, and not more than a moderate part in King Edward Street. It is all back land from the principal thoroughfares which surround the site of Christ's Hospital. The report placed in the hands of the govenors gives a full account of the negotiations which have been carried on since 1884. The one letter on which we have all along rested in confidence that sooner or later the governors of Christ's Hospital would carry out its promises is the letter of December 1st, 1893: "That, subject to the consent of the Charity Commissioners and to such restrictions as may be necessary with regard to the building line, negotiations be entered into with the governors of St. Bartholomew's Hospital to take as much of this hospital's London site as they may wish to have on the principle of arbitration in the usual way with an umpire." That was an engagement voluntarily entered into by the council of Christ's Hospital. On many occasions they have repeated their desire to deal with this hospital in such a way as to meet its requirements. Unfortunately, however, they have never gone any further than words, and they have never done anything that I am aware of to translate their goodwill and good wishes into action. I have here the notes of an interview between the Site Committee of Christ's Hospital and a deputation of this hospital; and at that interview several members of the Site Committee recognised to the fullest extent the moral obligation which rested on Christ's Hospital to deal with this hospital in the way set out in the letter I have read, of December 1st, 1893. The deputation took place in 1898, and a little later on the treasurer of Christ's Hospital, Mr. Alderman Morgan, wrote, "I agree that St. Bartholomew's Hospital have some ground of complaint for the way in which they have been treated." Later on, only so short a time ago as November 16th, 1899, in a letter which is set out in the report, the governors of this hospital, that is to say, Christ's Hospital, said they "have still but one desire, viz., whilst protecting the interests of Christ's Hospital to arrive at a friendly solution of the difficulty of negotiating as to the amount to be paid for such portion of this hospital's site as St. Bartholomew's Hospital may require." Well, gentlemen, what happened? Although we were advised that it was a doubtful course on our part to make an offer, still it was suggested to us, amongst others by His Royal Highness the President, that we had better make an offer. We consulted Mr. Vigers, a very eminent land surveyor, and on his advice we made an offer. Instead of their replying and doing anything to carry out their wishes that some friendly understanding should be arrived at, they simply tell us that they have received a much better offer, not for the portion of land we have been negotiating for, but for the whole site. Your Royal Highness and gentlemen, it seems to me that as a matter of public policy it is a very doubtful question whether a great public body like Christ's Hospital, dealing with another great body like this hospital, is acting quite in the way one would desire to see, in not considering the urgent and important public interests involved by the desire of this hospital to purchase land for its development, and I am bound to say, as it seems to me, that when gentlemen like the members of the Council of Christ's Hospital have voluntarily made an offer to this hospital, even though it is not binding in a court of law, there is a strong moral obligation upon those gentlemen, as honourable men, to adhere to their obligations. (Cheers.) Considering the numerous letters that have passed between us and the strong expressions of good will which have been made by the Council and the Sites Committee of Christ's Hospital, I have always thought there were solid grounds for expecting that the answer we should receive would be—"We are not able to accept the offer, but we shall be very glad to enter into negotiations with you so as to enable you to buy the land you require, and us to carry out our obligations with you." We have never received that help and that encouragement, and I am bound to say that I do not think the correspondence which has passed between us and Christ's Hospital shows either the great courtesy or consideration which one great public body should hold to another. (Hear, hear.) I have ventured to put very generally before you our position. I can assure you, gentlemen, and His Royal Highness, the President, I am sure, will believe that I speak in perfect sincerity, that there are a great many things in this hospital which require amendment. The hospital is by no means as perfect as we should like to see. It has been for many years the leading hospital in London, and it has been looked up to as being a hospital which should set an example of how things should be done. But what has been the difficulty? Not that we have not recognised the shortcomings of this hospital, not that we have not been most anxious to deal with them, but that we have simply been prevented by the absolute impossibility of providing land and space upon which to build. (Hear, hear.) I have seen in every hospital which has been constructed within recent times many things which I should like to see here. If His Royal Highness's Visiting Committee were to come here they would very likely say that there are many things that might be better than they are now, but I do not think the Visiting Committee would ever be able to say that we have not done our very best with the means at our disposal. I beg to thank your Royal Highness and gentlemen for listening to the remarks I have made. I think this is a question of very great public importance, and I trust that a reasonable, satisfactory, and rational conclusion between these two great bodies may be arrived at. (Cheers.)

Sir Sydney Waterlow said that as far back as the year 1885 the treasurer of Christ's Hospital wrote on behalf of his governing body, stating that they would not fail to confer with the governors of that hospital in reference to the portion of their land which St. Bartholomew's Hospital desired to acquire. In the year 1890, after the constitution of Christ's Hospital had been revised, the new governing body resolved that negotiations be entered into with the governors of St. Bartholomew's to take as much of the Christ's Hospital site as they wished to have on the principle of arbitration with an umpire. They thus had the old governing body and the new governing body agreeing to sell them the land by arbitration. If that had been an agreement between two honourable commercial men there could not be a doubt but that it would have been regarded as binding, morally if not legally, but the governors of Christ's Hospital, by their letter of November last, entirely repudiated their agreement of 1893, and refused to sell to them the land which they required for the enlargement of their hospital. The necessity for this enlargement was shown most clearly and distinctly by the letter from the medical and surgical staff dated November last, in which they practically asked them to buy the whole site of Christ's Hospital, and, personally, he was inclined to think that that would be the wisest course, but they were told by the report that this would be a very "speculative undertaking." Speaking, however, for himself, personally, while he quite agreed that their first course was to try to buy the land which they wanted, he could not resist the feeling that the purchase of the whole would not be a very speculative undertaking, and would ensure the development of the hospital to the extent required by the medical staff. He held in his hand the return of the sums available for hospital purposes during the last thirty years, after paying all expenses. In 1869 their net revenue was £38,462, and in 1899 £72,454, or an increase of £34,000, thus nearly doubling their income in thirty years in face of great losses sustained upon many thousand acres of agricultural property in Essex, Lincolnshire, Kent, and elsewhere. Remembering that so large a portion of their property consisted of houses and land in the great metropolis this increase was certainly worth at least thirty-three years' purchase, and this would add £1,132,000 to their property in thirty years. They could not close that discussion without assuming for a moment that Parliament might not give them compulsory powers to purchase the land they required, and that the governors of Christ's Hospital might not agree upon any terms that they could accept. What position would they be placed in? It seemed to him that they would have but two alternatives. One was to divide their work, separating to some extent the educational and scientific work from the nursing and treatment of sick patients. They possessed some acres of small houses about a quarter of a mile north of that hospital, where they might build a new hospital for some of the patients, and where they might carry on the out patients' work. That would be rather an advantage to the patients, as the new hospital would be nearer their homes. If this course were not thought desirable, there was another alternative. They might pull down all the older buildings, many of which were very dilapidated, and the four blocks containing the patients. They would, perhaps, have to give up the church, the official residences, and the ground around them, and by rebuilding the hospital on modern lines with all the best modern appliances they could, no doubt, increase the accommodation 30 to 40 per cent. There were, he knew, many medical men and sanitary authorities of the highest standing who thought that no buildings should be used certainly for more than a century for hospital purposes—their present wards were built more than 150 years ago, and the question arose whether they ought not, under any circumstances, to re-build them. Some of the governors might say, "But where is the money to come from?" He thought he had shown by his reference to the accounts that there would be no financial difficulty, and with the consent of the Charity Commissioners they might, having regard to their excellent security, borrow all the money they wanted, but should this fail them they ought never to forget that the public of the present day were never unwilling to subscribe to the relief of the sick poor of this great metropolis. (Cheers.) He concluded by reading the resolution, which was as follows: "That this Court, having considered the report of the treasurer and almoners upon their negotiations for the acquisition of a portion of the site of Christ's Hospital, concurs with the House Committee in approving and adopting the same. That the Court instructs the treasurer and almoners to adhere to the offer of £117,000 which has been made under competent advice; or, failing its acceptance, to continue to endeavour to effect a settlement on the basis of the resolution of the Council of Almoners of Christ's Hospital passed on 1st day of December, 1893, and communicated to this Court. That, if neither of the before mentioned alternatives result in a contract to purchase, the treasurer and almoners be empowered to take the necessary steps for obtaining from Parliament compulsory powers for the acquisition of the land that may be required." Practically, that resolution only committed them that day to a reference back to the treasurer and almoners, with increased power to act in the matter on their behalf. (Cheers.)

Mr. William Flux, a past almoner, seconded the resolution. He said that in 1819 that hospital parted to Christ's Hospital with a portion of the land which they were now desirous of reacquiring. There was recorded in the minute-book of their body a resolution from which he would read: "Resolved that in all proceedings between the two hospitals respecting the exchange of premises for the accommodation of Christ's Hospital, the governors of this hospital have been actuated by the most liberal motives, and an anxious desire of preserving the utmost harmony between the institutions by contributing to the convenience of that hospital as far as possible without manifest injury to their own." His impression was that their records gave a true view of what should be the state of things between Christ's Hospital and St. Bartholomew's Hospital at the present moment.

The Lord Mayor supported the resolution. He said that the discussion with regard to the bargain, and the honourable understanding which had been entered into between Christ's Hospital and St. Bartholomew's Hospital carried to his mind great weight, and he could not altogether appreciate the position that Christ's Hospital took up in this matter. Undoubtedly a distinct agreement and understanding was arrived at. Anything the Corporation could do to bring about an understanding, he need scarcely say, they would undertake to do with very great pleasure. (Cheers.)

Mr. G. Baker said that for the last thirty-two years he had been a governor of that hospital, and for the greater portion of that time he had served on the House Committee, and for the last seven years he had acted strenuously as almoner. He, therefore, knew something of the affairs of the hospital. Sir Sydney Waterlow had referred to the large increase of their income, but the large increase was very greatly due to the falling in of building leases; they therefore could not expect to have that same increase in the future. In May and June, 1898, he was asked by the treasurer to accompany him and the clerk as a deputation to the site committee of Christ's Hospital. The first suggestion that they had to make—and it came directly from them—was that they should take the whole site. (Hear, hear.) They impressed that upon them, and argued that if they did not want the whole site at the present time it would be sure to be useful in the future, and that probably they could let off a large portion on favourable terms. All but one on that site committee acknowledged that they were morally bound by the resolution of 1897, but that at the same time it was suggested that the almoners of 1893 were not the same as those of 1898, and that some of those of 1898 held different views. One modern almoner said that he was not a party to the resolution of 1893, and that he should oppose the mode of settlement suggested by it. He ventured to recommend to the notice of the almoners of the site committee of Christ's Hospital the words that fell from Lord Salisbury, but ten days ago, *apropos* of the Cromwell statue. His words were: "It seems a matter not only of Common Law, but of common honesty, that when a First Commissioner of Works is changed, his successor shall own himself bound by the promise of his predecessor." (Cheers.) Next, they had heard that they had called in Mr. Vigers for advice. In a very long interview with him after he presented his report, they especially asked him as to the purchase of the whole site, and whether it would be possible to create ground rents, giving up the whole of the frontage and reserving the back land for the hospital. His advice was most emphatic. He said: "Buy only what you want; to take the whole quantity would be to enter into a building speculation which very likely would have disastrous results. There is no great demand for ground in the Newgate Street area, and it is very likely that it would be ten years before you would get the ground covered." In the face of that advice, he did not see how they could possibly purchase the whole site.

The Prince of Wales: My duty, as occupying the chair as president of this great institution, is to put the resolution which has been so ably moved by Sir Sydney Waterlow and seconded by Mr. Flux. Before doing so, let me assure you of the deep interest I shall always take in the concerns of this ancient institution, with which I have been connected now for so many years. (Cheers.) Indeed, the treasurer, in the able speech he has made, has not in any way exaggerated in what he kindly said about me. I feel the position of this hospital at the present moment, and it cannot be denied that it is a very serious one. As it has been fully explained to you, although everything, so far as it goes, is in a highly satisfactory state, still, owing to the want of space we are not able to meet the exigencies of the time and follow the lead of other hospitals so far as great scientific improvements and inventions are concerned, which every year are coming more and more to the front. I looked upon it as a matter of absolute necessity that we should acquire this one and a half acres of land. Honestly, gentlemen, I tell you it is my own private opinion that I should be glad to see us obtain the whole site. (Cheers.) But we have to cut our coat according to our cloth; and I only wish that we had more cloth from which to be able to cut a better coat. However, from what I have heard, that is impossible. Even now we are in considerable doubt as to whether we shall get even the part we want. I do not know why Christ's Hospital have not thought fit to stick to their original engagement with us, and I cannot help feeling, and deeply regretting, as I am sure you all do, the scant courtesy, to say the least of it, with which they have treated us. Gentlemen, we are all busy men; you are busy and I am busy, and I look upon this as a business meeting and not one for oratory, however much we should like to display the powers we possess in that direction. Unless any governor wishes to say anything, which of course we should be only too glad to hear, I should put the resolution to the meeting. We have had some very able speeches, and I think all the salient points have been thoroughly dealt with. As it is not possible for us to acquire the whole site, as we have not the means of doing so, anyhow at present, I hope this resolution which has been placed before you will be carried. I also trust that we shall be backed up by the Charity Commissioners, and that Parliament at least will not refuse what is for the benefit of this public institution, which is one of the oldest and one of the most important institutions of its kind. It is perfectly impossible to provide everything absolutely necessary unless we have more space, and I am sure we all earnestly hope that this additional space may be acquired. With these few words I have great pleasure in now putting the resolution.

The resolution was then put and carried unanimously.

The proceedings then terminated.

### CENTRAL LONDON THROAT AND EAR HOSPITAL.

Captain Alfred Hutton presided at the twenty-sixth annual meeting of the Central London Throat and Ear Hospital on Tuesday, 26th ult. Mr. Kershaw, the secretary, read the report, which stated that during 1899 the in-patients numbered 272, the average stay being 14 days. The in-patients were just four times as many as in the first year the wards were open—a difference accomplished by lessening the number of days of residence, though not by admitting less serious cases. The out-patients again largely exceeded those of previous years, numbering 8,843, with 55·112 attendances. The ordinary income for the year was £2,474, as compared with £2,406 in the previous year. Of this, no less than £1,792 was from the voluntary contributions of patients. But although the aggregate is a large sum, the individual contribution is merely nominal, averaging but a few pence per visit. The ordinary expenditure was £2,224, and the extraordinary expenditure £403. The average cost of each in-patient (14 days) worked out at £2 14s. 11d., and of each out-patient (six attendances) 3s. 4d.—figures which would bear comparison with those of the larger general hospitals. The council of the Prince of Wales's Fund had awarded them, after a visit of inspection, £150 towards the cost of a new operating-room. Deep regret was expressed at the death during the year of Mr. Van Tromp, who, since 1881, had been vice-chairman of the committee. General Stewart had resigned his seat on the general committee owing to his prolonged absence from town, and Mr. Carmichael Thomas had been appointed a member of that body. Mr. Lennox Browne, one of the two surviving founders of the hospital, after 25 years of service, had resigned the office of senior surgeon, but had accepted the office of consulting surgeon, and would continue to be vice-chairman of the General Committee and chairman of the Medical Committee. Mr. W. Hotten George had been appointed anæsthetist. Mr. Thomas proposed, and Dr. Dundas seconded, the adoption of the report, which was carried. The committee was re-elected, with the addition of Mr. Calkin Lewis, it being decided to hold meetings every two months, instead of three as hitherto. Captain Hutton was re-elected chairman and treasurer. Dr. Dundas Grant referred in warm terms to the retirement of Mr. Lennox Browne, and moved a vote of thanks to him for his services. This was carried by acclamation, and the proceedings terminated.

